# Cocaine Potentiates Astrocyte Toxicity Mediated by Human Immunodeficiency Virus (HIV-1) Protein gp120

**DOI:** 10.1371/journal.pone.0013427

**Published:** 2010-10-15

**Authors:** Yanjing Yang, Honghong Yao, Yaman Lu, Chao Wang, Shilpa Buch

**Affiliations:** Department of Pharmacology and Experimental Neuroscience, University of Nebraska Medical Center, Omaha, Nebraska, United States of America; Boston University School of Medicine, United States of America

## Abstract

It is becoming widely accepted that psychoactive drugs, often abused by HIV-I infected individuals, can significantly alter the progression of neuropathological changes observed in HIV-associated neurodegenerative diseases (HAND). The underlying mechanisms mediating these effects however, remain poorly understood. In the current study, we explored whether the psychostimulant drug cocaine could exacerbate toxicity mediated by gp120 in rat primary astrocytes. Exposure to both cocaine and gp120 resulted in increased cell toxicity compared to cells treated with either factor alone. The combinatorial toxicity of cocaine and gp120 was accompanied by an increase in caspase-3 activation. In addition, increased apoptosis of astrocytes in the presence of both the agents was associated with a concomitant increase in the production of intracellular reactive oxygen species and loss of mitochondrial membrane potential. Signaling pathways including c-jun N-teminal kinase (JNK), p38, extracellular signal-regulated kinase (ERK)/mitogen-activated protein kinases (MAPK), and nuclear factor (NF-κB) were identified to be major players in cocaine and gp120-mediated apoptosis of astrocytes. Our results demonstrated that cocaine-mediated potentiation of gp120 toxicity involved regulation of oxidative stress, mitochondrial membrane potential and MAPK signaling pathways.

## Introduction

AIDS or acquired immunodeficiency syndrome is a multisystem disorder involving the central nervous system (CNS). Human immunodeficiency virus type 1 (HIV-1) infection of the brain leads to a range of neurodegenerative complications, often referred to as HAND [1]. The most severe form of HAND, HIV-associated dementia (HAD), is characterized by neuropathological changes that include reactive astrocytosis and formation of microglial nodules and multinucleated giant cells in a setting of enhanced virus replication and/or cellular activation [2]. While CD4 lymphocytes and microglia/macrophages are the major target cells for HIV-1 infection [3], limited infection has also been observed in astrocytes [4].

Astrocytes, the major non-neuronal cells in the brain, have been implicated in providing tropic support for the neurons. Typically these functions include segregation, maintenance, and support of neurons, including clearance and release of extracellular glutamate [5], [6], scavenging of oxygen free radicals [7] and homeostatic maintenance of extracellular ionic environment and pH [8], [9] These functions provide metabolic substrates for neurons [10], and couple cerebral blood flow to neuronal activity [11]. It is thus plausible to envision that dysregulation of astrocyte-specific functions, can critically impact neuronal survival [12]. It has been shown that progression of HAD is also accompanied by apoptosis of astrocytes [13], [14], [15]. Significantly, greater numbers of apoptotic astrocytes have been detected in the brains of HIV/AIDS patients with rapidly progressing dementia [16], and detection of apoptotic astrocytes appeared to be more common in patients with dementia, compared to non-demented HIV/AIDS patients [14], suggesting a role for astrocytic cell loss in the neuropathogenesis of HAD.

Mounting evidence suggests that drugs of abuse such as cocaine can accelerate the incidence and progression of HAD [17], [18], [19]. For example, drug-abusing HIV-1-positive individuals exhibit more severe cognitive impairment compared with the non-drug-abusing HIV-positive counterparts. Despite the recognized impact of drugs of abuse on the clinical course of HIV-associated brain pathology, little is known about mechanisms that underlie the ability of these drugs to enhance toxic effects of HIV-1 proteins in the brain. Emerging concepts suggest that psychostimulants such as cocaine can have profound effects on the susceptibility of neuronal cells to HIV-1 virotoxins. However, whether cocaine can also potentiate the toxic effect of HIV-1 gp120 on astrocytes remains unclear.

In the present study, we hypothesized that increased progression and incidence of HAD-associated astrocytic cell death in drug-abusing HIV-infected individuals could be attributed to the potentiation by cocaine of gp120-induced apoptosis. In this report we have dissected the converging signaling pathways including the c-jun N-teminal kinase (JNK), p38 and extracellular signal-regulated kinase (ERK)/mitogen-activated protein kinase (MAPK) pathways and the downstream transcription factor NF-κB, leading to cocaine and gp120-mediated apoptosis of astrocytes.

## Materials and Methods

### Animals

Pregnant female Sprague-Dawley rats were purchased from Charles River Laboratories, Inc. (Wilmington, MA). All of animals were housed under conditions of constant temperature and humidity on a 12-h light, 12-h dark cycle, with lights on at 0700 h. Food and water were available ad libitum. All animal procedures were performed according to the protocols approved by the Institutional Animal Care and Use Committee of the University of Nebraska Medical Center and National Institute of Health.

### Reagents

Viral gp120 (IIIB strain) was a gift from Marcus Kaul (Burham Institute for Medical Research), gp120 (Bal) was obtained the AIDS Research and Reference Reagent Program of National Institutes of Health. The rationale for choosing gp120 IIIB is based on the utilization of CXCR4 co-receptor in astrocytes. Quantification of gp120 in the CNS or CSF has been difficult because of the limited cross-reactivity of anti-gp120 Abs. The relevance of using 200 ng/ml (or 1670 pM) of gp120 in our study is based on the reports that levels as high as 796 ng/ml of gp120 have been detected in HIV-infected subjects during early infection [20] and furthermore, that these concentrations have also been used in earlier reports for neuronal toxicity by others and us [21], [22], [23]. Additionally, Brenneman et al have also demonstrated gp120 neurotoxicity in mouse hippocampal neuronal-glial cell cultures at concentrations ranging from below 1 pM to 1 nM [24]. The specific MAPK/ERK kinase (MEK) 1/2 inhibitor U0126, p38 inhibitor SB203580, and JNK inhibitor SP600125 were purchased from Calbiochem (San Diego, CA). Nuclear factor (NF)-κB inhibitor l-1-tosyllamide-2-phenylethyl chloromethyl ketone (TPCK) was purchased from Sigma Chemicals (St. Louis, MO).

#### Primary astrocyte cultures

Rat primary astrocytes were prepared according to the previously described protocol with slight modifications [25]. Briefly, tissues from whole brains of postnatal (P1–P2) Sprague-Dawley rats were triturated and cells were plated on poly-D-lysine pre-coated cell culture flasks in Dulbecco's modified Eagle's medium containing 10% fetal bovin serum, 100 U/ml penicillin and 100 µg/ml streptomycin. Cultures were maintained at 37°C in a humidified atmosphere of 5% CO_2_/95% air. After reaching a confluent monolayer of glial cells (10–14 days), microglia were separated from astrocytes by shaking off. The enriched astrocytes were >96% positive for glial fibrillary acidic protein when assessed by immunocytochemical staining.

#### MTT assay

Cell viability was measured by 3-(4,5-dmethylthiazol-2-yl)-2,5-diphenyl Tetrazolium bromide (MTT) method. Briefly, cells were collected and seeded in 96-well plates. Different seeding densities were optimized at the beginning of the experiments. Rat primary astrocytes were exposed to fresh medium containing various concentrations of cocaine (1, 10, 100 µM) with or without 200 ng/ml gp120. After incubation for up to 48 h, 20 µl MTT tetrazolium salt dissolved in Hank's balanced salt solution at a final concentration of 5 mg/ml was added to each well and incubated in CO_2_ incubator for 4 h. Finally, the medium was aspirated from each well and 200 µl of dimethyl sulfoxide was added to dissolve the formazan crystals and the absorbance of each well was obtained using a plate counter at test and reference wavelengths of 570 nm and 630 nm, respectively. All of experiments were repeated at least four times.

#### Immunocytochemistry

Cells were fixed in 4% paraformaldehyde, followed by blocking with phosphate-buffered saline (PBS) containing 10% normal goat serum. After blocking, cells were incubated at 4°C overnight with the anti-cleaved caspapse-3 antibody (Cell signaling, Danvers, MA). Following washes, cells were then incubated with the secondary goat anti-rabbit Alexa Fluor 488-conjugated antibody (1∶500). For negative controls, cells were treated as described above, except that the primary antibody treatment was omitted. All experiments were repeated at least three times.

#### Reactive oxygen species (ROS) assay

Intracellular production of ROS was measured by 2′,7′-dichlorfluorescein diacetate (DCFH-DA) oxidation. Primary astrocytes were treated with cocaine and/or gp120 for 1 h and then incubated with DCFHDA (Sigma, St. Louis, MO) at 20 µM for 30 min. After incubation, cells were washed with PBS and the fluorescence was visualized immediately at wavelengths of 485 nm for excitation and 530 nm for emission using an inverted fluorescence microscope. Total green fluorescence intensities in every well were quantitated using NIH Image J software. All of experiments were repeated at least three times.

#### Analysis of mitochondrial membrane depolarization

The change in mitochondrial membrane potential in the astrocytes was monitored using the mitochondrial membrane potential detection kit (Cayman Chemical Company) according to the manufacturer's instructions. Briefly, rat primary cultured astrocytes cultured in either 24-well plate (1×10^5^ cells per well) or 96-well plate (3×10^4^ cells per well) were treated with gp120 and/or cocaine followed by treatment with 1×JC-1 reagent diluted in serum-free culture medium for 20 min at 37°C in 5% CO_2_. Thereafter, cells were rinsed once in 1× rinsing buffer provided in the kit. Uptake of the dye was then assessed using the Nikon inverted fluorescence microscope TE2000-E (Nikon, Tokyo, JAPAN). Fluorescence was also measured using the FL600 fluorescent plate reader (Bio-Tek Instruments, Winooski, VT) at the excitation wavelengths of 485 nm and 535 nm. Fluorescence intensity also was quantified using NIH Image J software. All experiments were repeated at least three times.

#### Western blotting

Treated cells were lysed using the Mammalian Cell Lysis kit (Sigma, St. Louis, MO) and the NE-PER Nuclear and Cytoplasmic Extraction kit (Pierce, Rockford, IL). Equal amounts of the corresponding proteins were electrophoresed in sodium dodecyl sulfate polyacrylamide gels (12%) under reducing conditions followed by transfer to PVDF membranes. Blots were blocked with 5% nonfat dry milk in phosphate buffered saline and probed with antibodies recognizing the phosphorylated forms of JNK, p38, ERK1/2 (Cell Signaling, Danvers, MA; 1∶500), cleaved Caspase-3 (Cell Signaling, Danvers, MA; 1∶500) and NF-kB p65 (Cell Signaling, Danvers, MA; 1∶1000). The secondary antibodies were alkaline phosphatase-conjugated to goat anti mouse/rabbit immunoglobulin G (IgG) (1∶5000). Signals were detected by chemiluminescence (Pierce, Rockford, IL).

#### Statistical analysis

Data were expressed as mean±SD. Significance of differences between control and cocaine-treated samples was determined by one-way ANOVA followed by the post hoc Least-Significant-Difference (LSD) test. Values of *p*<0.05 were considered to be statistically significant.

## Results

### Cocaine exposure resulted in enhanced gp120 toxicity in rat primary astrocytes

**Figure 1:**
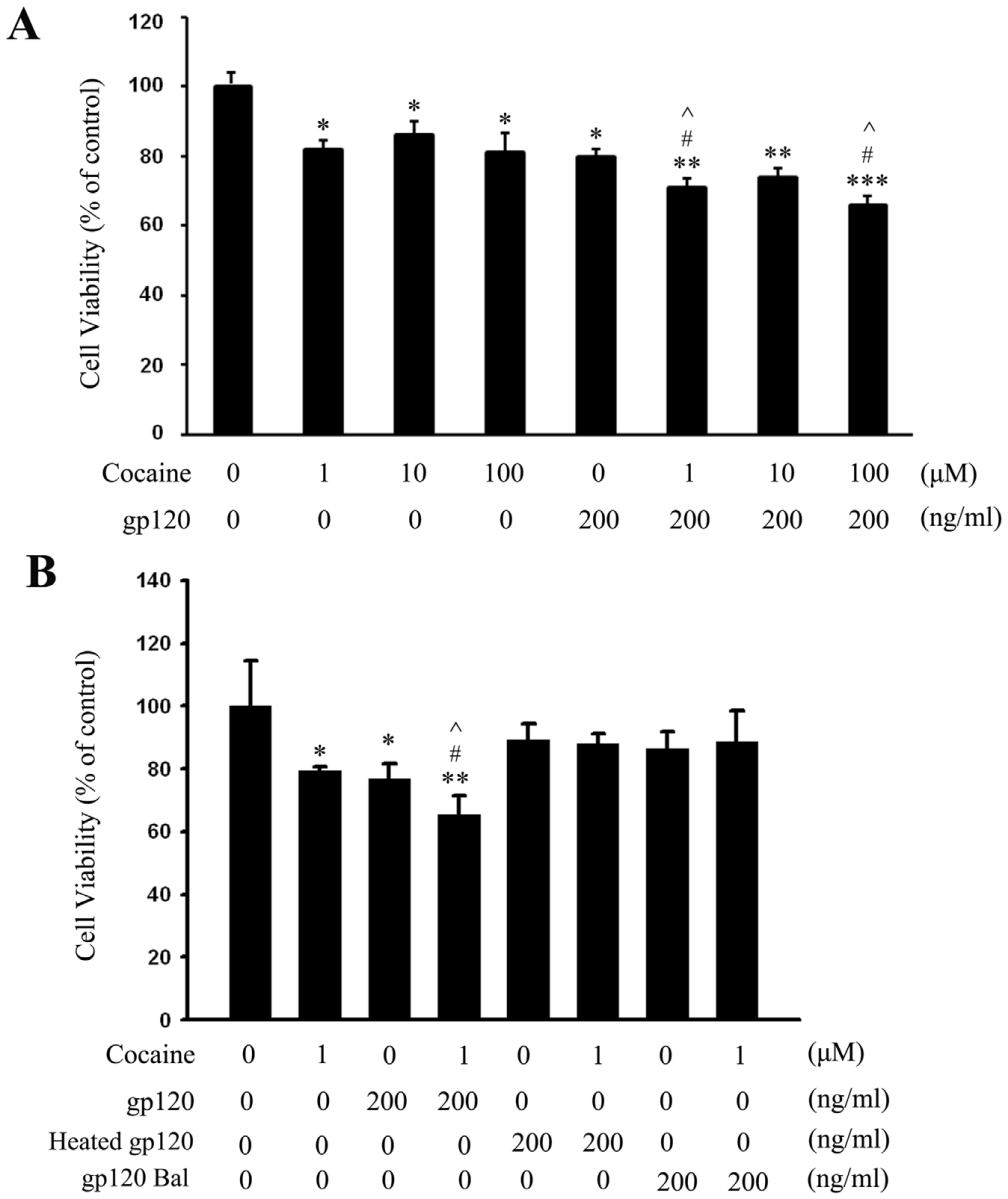
Effects of cocaine and/or gp120 on cell viability in rat primary astrocytes. (A) Effects of 1, 10, 100 µM cocaine in the presence or absence of gp120 IIIB (200 ng/ml) for 48 h on the survival of rat primary astrocytes using the MTT assay. (B) Effects of gp120 IIIB, heated inactivated gp120 IIIB and gp120 Bal (200 ng/ml) on the survival of rat primary astrocytes using the MTT assay. All the data are presented as mean ± SD of four individual experiments. *p<0.05; **p<0.01; ***p<0.001 versus control group; # p< 0.05 versus cocaine group; ∧ *p*< 0.05 versus gp120 group.

To explore the toxic effects of cocaine and gp120, rat primary astrocytes were exposed to varying concentrations of cocaine (1, 10, 100 µM) with or without gp120IIIB (200 ng/ml) for 48 h followed by assessment of cell viability using the MTT assay. The rationale for choosing the above mentioned range of cocaine was based on the following reports. In human volunteers, plasma cocaine concentrations following intranasal administration of 1.5 mg/kg cocaine often range between 0.4 to 1.6 µM [26], while plasma cocaine concentrations in tolerant abusers, reach levels of up to 13 µM [27]. Additionally, cocaine concentrations of up to 100 µM and higher have been reported in postmortem brains of chronic human cocaine users following acute intoxication [28]. Based on these reports, we rationalized that cocaine concentrations ranging from 1 to 100 µM would be comparable to levels observed in humans. As shown in Figure 1A, increasing concentrations of cocaine (1, 10, 100 µM) resulted in decreasing cell viability by 18% (p<0.05), 14% (p<0.05), and 20% (p< 0.001), respectively. Interestingly, exposure of cells to 1 µM cocaine resulted in reduction of cell viability by 18%; gp120 alone, as expected, decreased cell viability by 20%, and both gp120 and cocaine together caused a statistically significant decrease in cell viability by about 29% (*p*<0.05 versus cocaine or gp120). On the other hand, heat-inactivated gp120 or CCR5-utilizing strain of gp120 (Bal) failed to exert toxicity on astrocytes (Figure 1B), thereby underpinning the specificity of gp120 IIIB toxicity on astrocytes.

### Cocaine enhanced gp120-mediated activation of caspase-3

**Figure 2:**
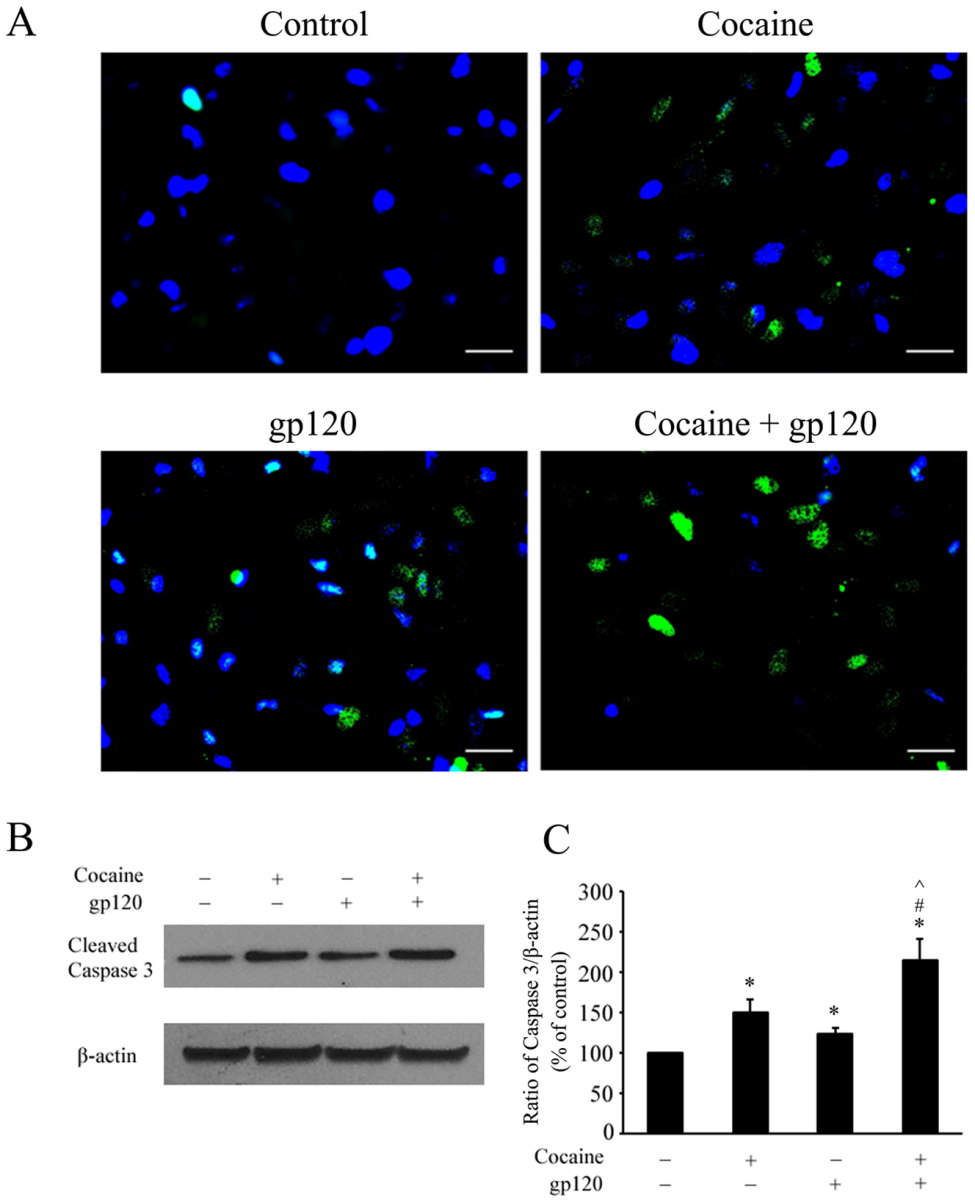
Effects of cocaine and/or gp120 exposure on caspase-3 activation in rat primary astrocytes. (A) Rat primary astrocytes treated with cocaine (10 µM) and/or gp120 IIIB (200 ng/ml) for 24 h were monitored for active caspase-3 by immunostaining using anti-cleaved caspase-3 antibody. Scale bars indicate 20 µm. (B) Rat primary astrocytes were treated with cocaine and/or gp120 for 24 h, followed by cell lysis and detection of anti-cleaved caspase-3 protein on a Western blot. (C) Densitometry scans of the ratio of band intensities of cleaved caspase-3/β-actin from three separate experiments. * p<0.05 versus control group; # p< 0.05 versus cocaine group; ∧p< 0.05 versus gp120 group.

To corroborate the findings that cocaine and gp120-mediated toxicity involved the apoptotic pathway, we next sought to investigate activation of caspase-3. As shown in Figure 2A, astrocytes treated with either gp120 or cocaine for 24 h demonstrated positive staining for activated caspase-3. Simultaneous treatment of astrocytes with both the agents resulted in further enhancement of caspase-3 positivity. These findings were further validated by Western blot analysis for activated caspase-3. As shown in Figure 2B and 2C, astrocytes treated with either gp120 or cocaine alone demonstrated a significant increase in expression levels of activated caspase-3 compared with untreated control cells (*p*<0.05 versus control). Treatment of astrocytes with a combination of both gp120 and cocaine, however, further increased the levels of activated caspase-3 compared with cells exposed to either agent alone (*p*<0.05 versus cocaine or gp120).

### Cocaine and gp120-mediated alterations in intracellular reactive oxygen species (ROS)

**Figure 3:**
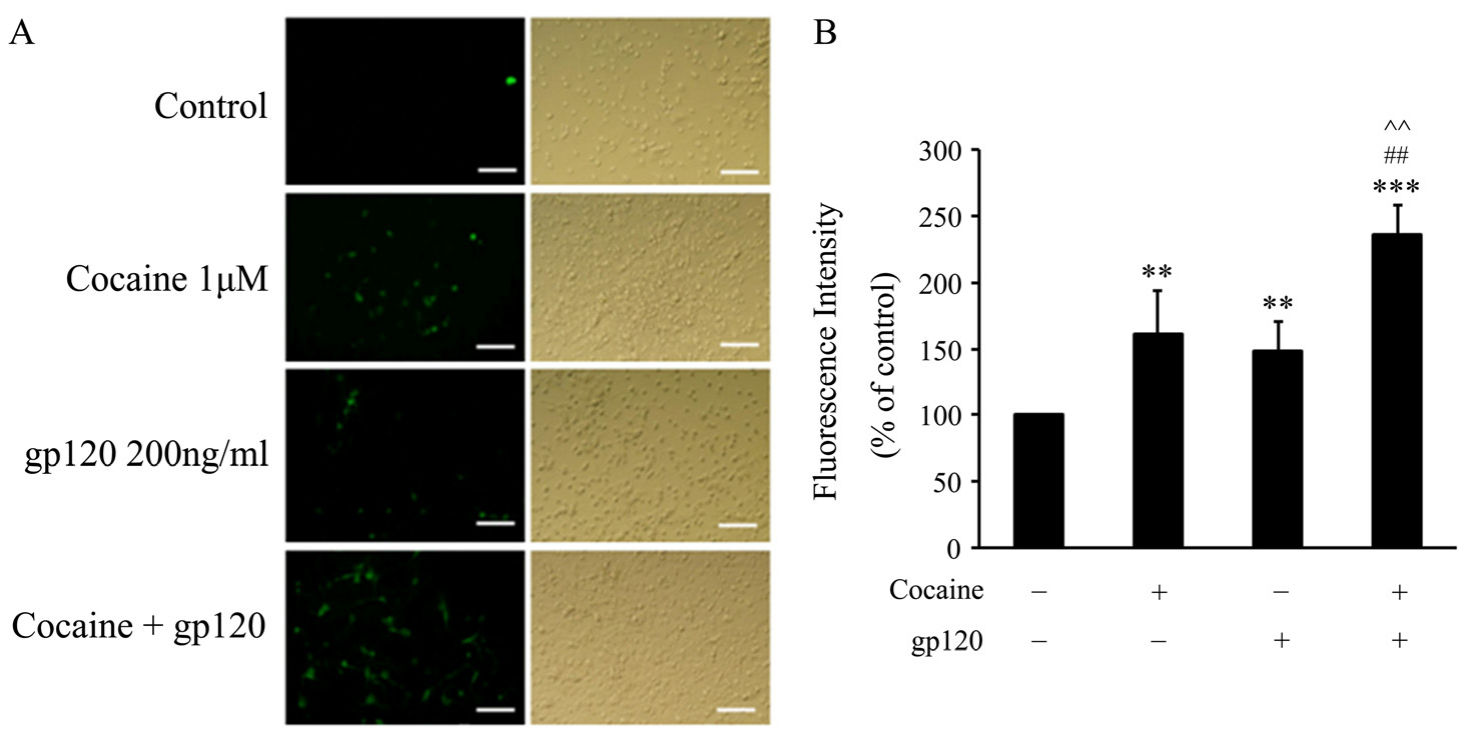
Effects of cocaine and/or gp120 on intercellular ROS production in rat astrocytes. (A) Cells treated with cocaine in the absence or presence of gp120 for 1 h were assessed for production of ROS using DCFH-DA assay. Treatment of astrocytes with cocaine and/or gp120 resulted in increased ROS production. Scale bars indicate 200 µm. (B) Quantification of ROS fluorescence intensity in cells treated with gp120 and/or cocaine using fluorescence plate reader. All the data are presented as mean ± SD of four individual experiments. ** p<0.01; ***p<0.001 versus control group; ## p< 0.01 versus cocaine group; ∧∧ p< 0.01 versus gp120 group.

ROS production plays a critical role in the mitochondrial dysfunction and subsequent cell death. We next sought to explore whether cocaine-mediated enhancement of the gp120 toxicity also involved production of ROS. Briefly, rat primary astrocytes were treated as described above followed by measurement of intracellular ROS using the DCHF-DA oxidation assay. As shown in Figures 3A and B, treatment of astrocytes with cocaine or gp120 alone resulted in increased intracellular ROS production was increased to 161% and 148% in the presence of cocaine and gp120 respectively, compared with untreated cells (*p*<0.01). This effect was augmented to 236% in the simultaneous presence of both cocaine and gp120 (*p*<0.001 versus control, *p*<0.05 versus cocaine or gp120 alone).

### Cocaine and gp120-mediated alterations in mitochondrial membrane potential

**Figure 4:**
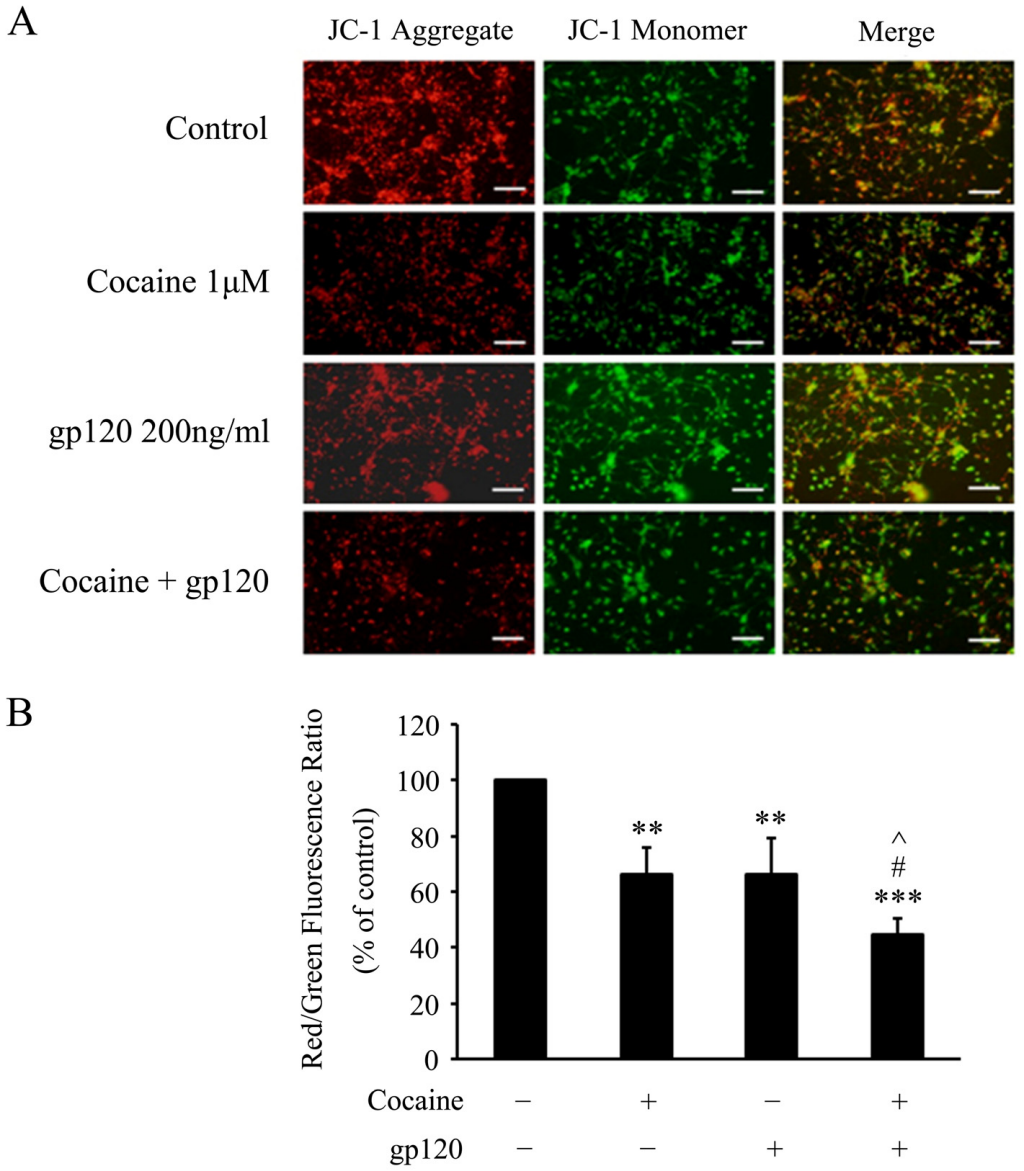
Effects of cocaine and/or gp120 on mitochondrial membrane potential in rat astrocytes. (A) Cells treated with cocaine and/or gp120 for 18 h were assayed for mitochondrial membrane potential by staining with JC-1 dye. Treatment with cocaine/gp120 resulted in reduction of the aggregation of JC-1 dye in the mitochondria (red fluorescence) and decreased ratio of the aggregate (red fluorescence) to monomer JC-1 (green fluorescence) in the cells. Scale bars indicate 200 µm. (B) Quantification of Δψm expressed as a ratio of J-aggregate to JC-1 monomer (red∶ green) fluorescence intensity. All the data are presented as mean±SD of three individual experiments. ** p<0.01; ***p<0.001 versus control group; #p< 0.05 versus cocaine group; ∧p< 0.05 versus gp120 group.

Mitochondria are a link between the initial insult stimuli and the initiation of apoptosis. Furthermore, since loss of mitochondrial membrane potential (Δψm), a consequence of mitochondrial membrane permeability is detected in several models of apoptosis, we sought to detect whether cocaine and/or gp120-mediated apoptosis involved change in ψm in astrocytes (Figure 4). Astrocytic mitochondrial membrane depolarization was assessed following exposure of cells to cocaine and/or gp120 for 18 h using JC-1 probe, which is a fluorescent lipophilic cationic dye that accumulates in mitochondria in proportion to the Δψm, that normally exists across the inner mitochondria membrane [29]. As shown in Figures 4A and 4B, there was increased membrane depolarization in astrocytes exposed to either gp120 or cocaine alone by 66% or 67%, respectively (*p*<0.01 versus control), and this effect was further enhanced in the presence of both cocaine and gp120 by 45% (*p*<0.05 versus cocaine or gp120 alone).

### Cocaine and gp120-mediated MAPK phosphorylation in astrocytes

**Figure 5:**
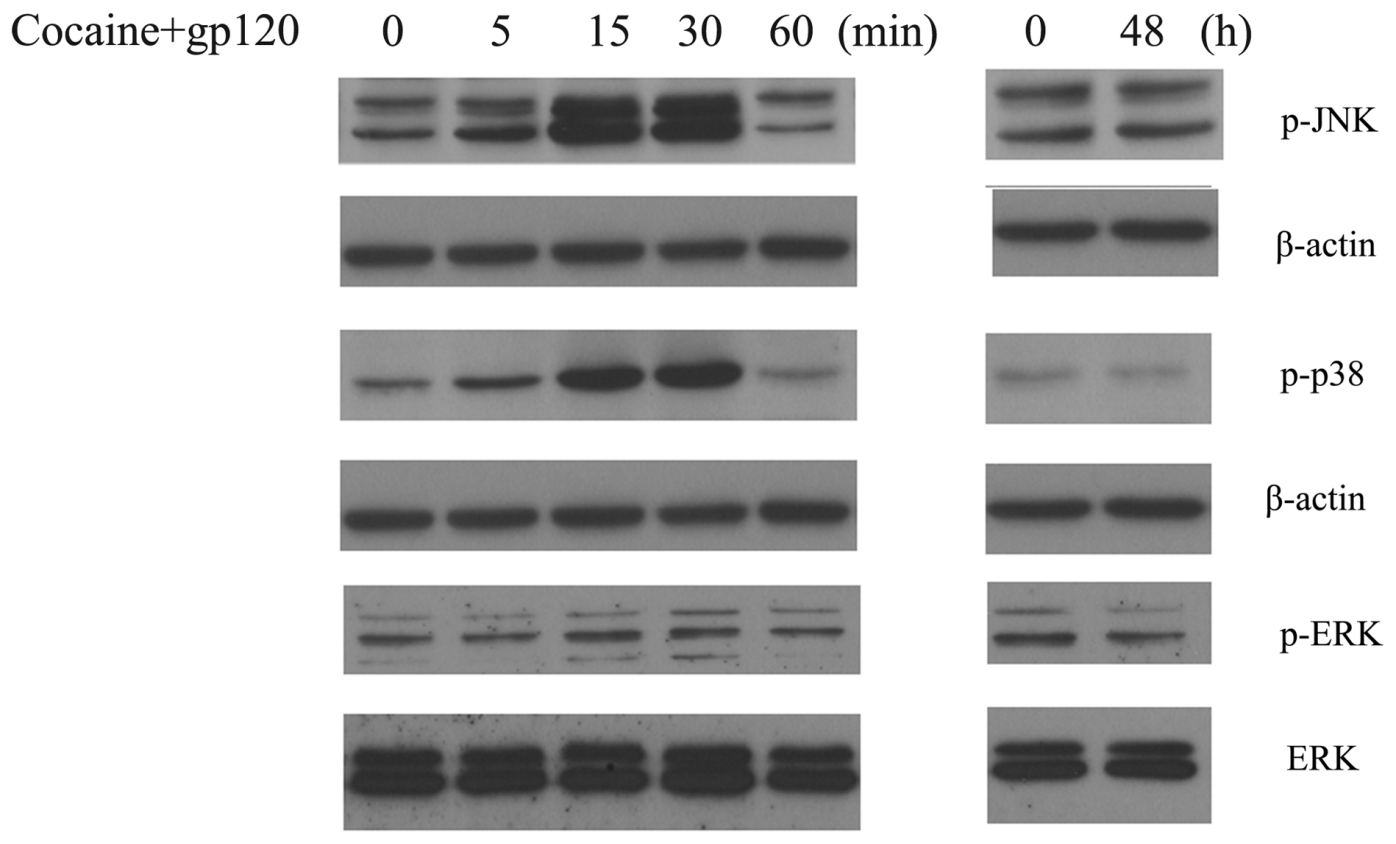
Cocaine and gp120 induced phosphorylation of MAPKs. Western blot analysis of time-dependent activation of ERK, JNK and p38 kinases in astrocytes treated with cocaine and gp120. Representative immunoblots are presented from 4 separate experiments.

Increasing evidence indicates that MAPK pathway plays an important role in apoptosis [30]. We next sought to investigate whether cocaine and/or gp120-mediated astrocyte apoptosis involved activation of MAPK signaling mediators. Cell lysates from astrocytes treated with cocaine and/or gp120 were assessed for activation of MAPK pathway signaling proteins using Western blot. Treatment of astrocytes with both cocaine and gp120 resulted in a transient time-dependent increase in phosphorylation of JNK, p38 and ERK (Figure 5) with maximal activation at 15 min post-treatment and a decline thereafter that was sustained even at 48 h compared with control cells (Figure 5).

**Figure 6:**
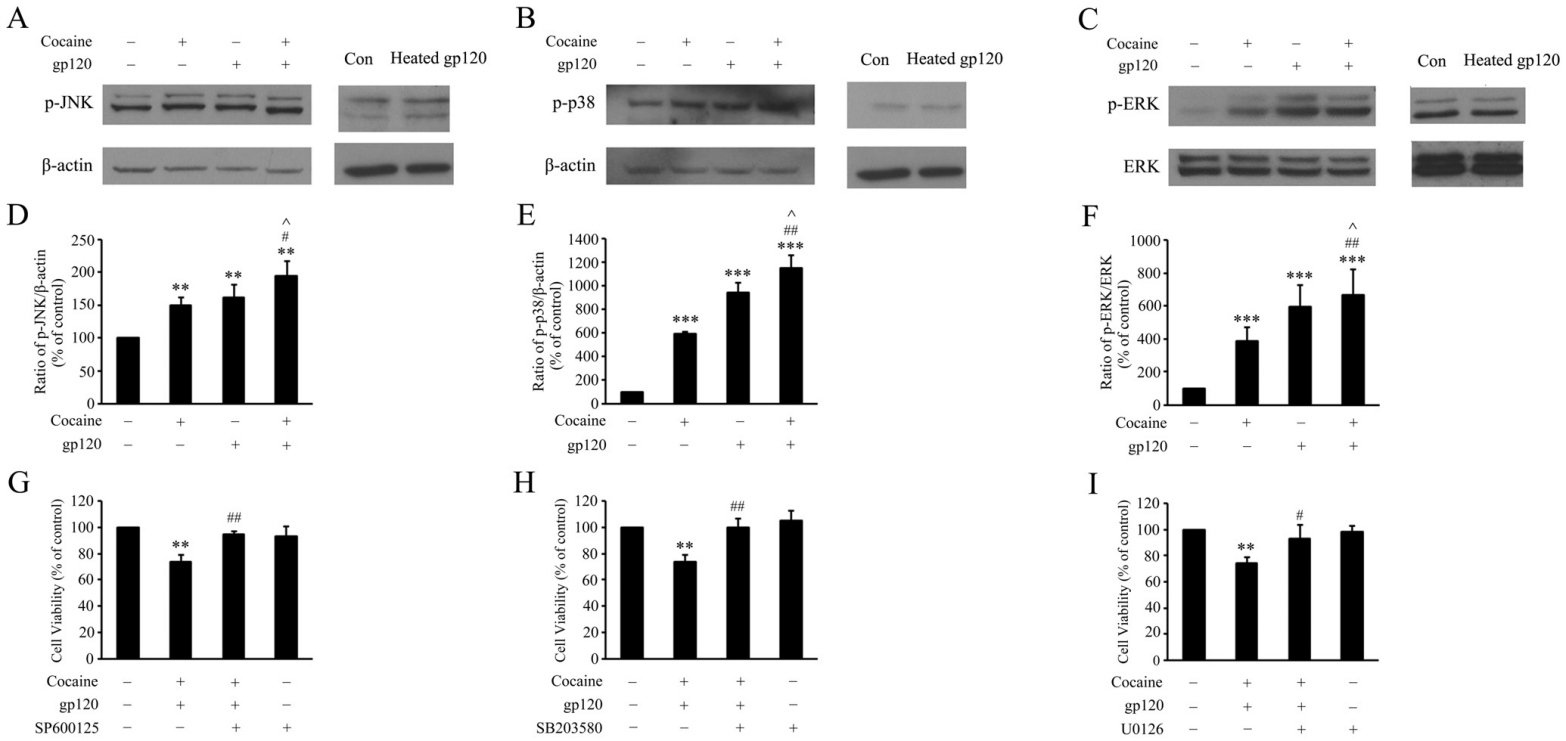
Involvement of MAPK pathway in cocaine and/or gp120-induced toxicity in rat primary astrocytes. (A–C) Western blot analysis of cytosolic lysates from cocaine (10 µM) and/or gp120 IIIB (200 ng/ml) or heat-inactivated gp120 IIIB)-treated cells (15 min) using antibodies specific for the phosphorylated forms of JNK, p38, ERK/MAPK respectively. (D–F) Densitometric analyses of phos-JNK/β-actin, phos-p38/β-actin, phos-ERK/ERK from three separate experiments. Representative immunoblot and the densitometric analysis of phos-JNK/β-actin, phos-p38/β-actin, phos-ERK/ERK from three separate experiments is presented. All the data in these figures are presented as mean±SD of three individual experiments. **p<0.01; ***p<0.001 versus control group; #p< 0.05, ## p<0.01 versus cocaine group; ∧ p<0.05 versus gp120 group. (G–I) Pharmacological inhibition of the MAPK pathway in astrocytes resulted in abrogation of cocaine and gp120-induced toxicity. All the data are presented as mean±SD from three individual experiments. **p<0.01 versus control group. #p<0.05, ##p<0.01 versus cocaine + gp120 group.

As shown in Fig 6A–C, there was increased activation of JNK, p38 and ERK1/2, in the presence of cocaine or gp120 alone and, this effect was further enhanced in the presence of both the agents. As expected, heat-inactivated gp120 failed to induce gp120-mediated phosphorylation of these MAPKs. The specificity of these MAPKs in the enhancement of cell toxicity was further confirmed by pre-treating the cells with pharmacological inhibitors specific for the respective signaling pathways (Figure 6 D–F). As shown in the Figures 6 (G–I), pretreatment of cells with JNK (SP600125; 20 µM), p38 (SB203580; 20 µM), or MEK1/2 (U0126, 20 µM) inhibitors for 1 hr followed by treatment with cocaine and gp120 resulted in amelioration of cell toxicity, further confirming the role of these pathways in the co-operative toxicity induced by these agents.

### Cocaine and gp120-mediated NF-κB increase in astrocytes

**Figure 7:**
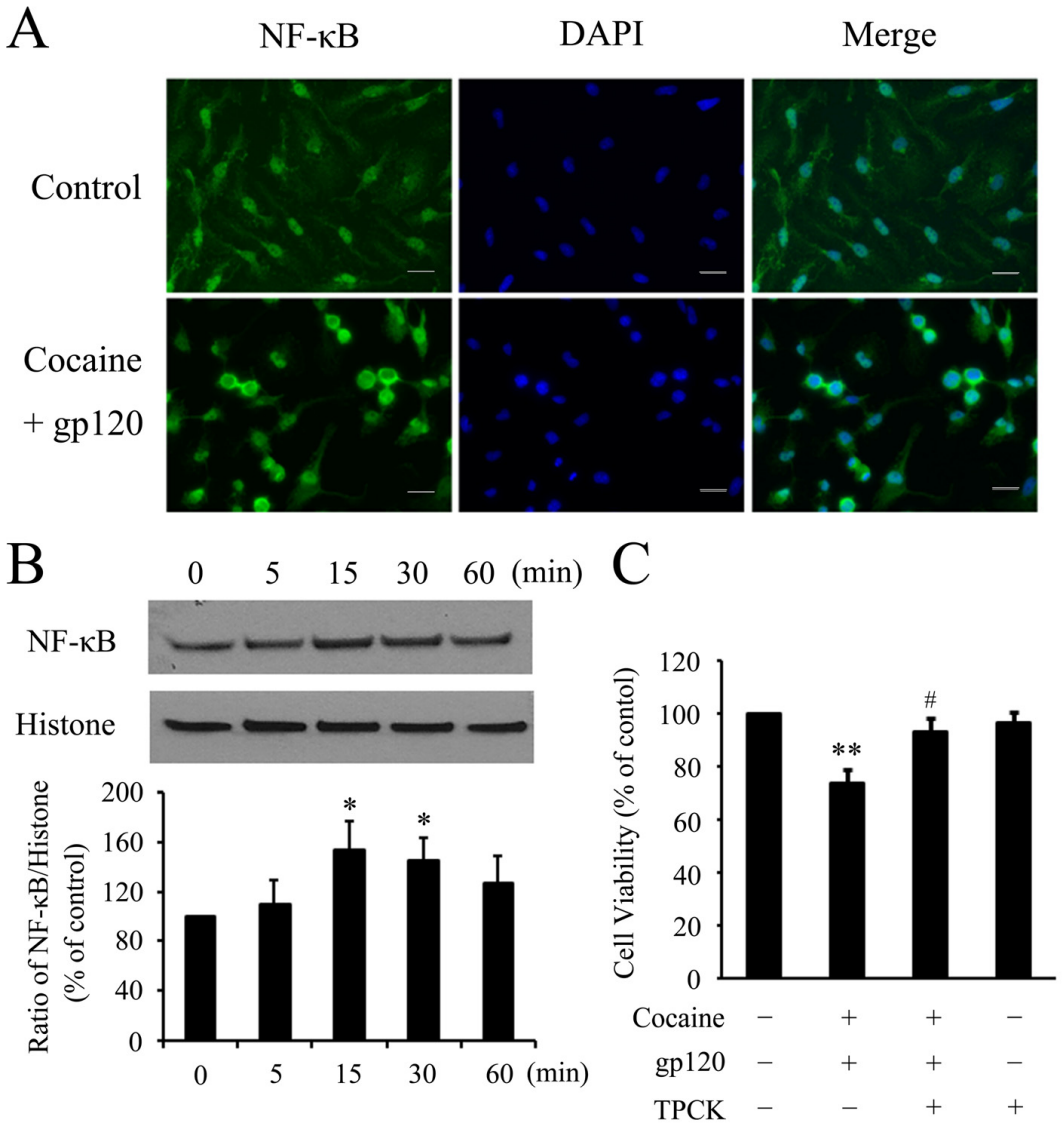
NF-κB is involved in cocaine and gp120-induced astrocyte apoptosis. (A) Rat primary astrocytes grown on coverslips were treated with cocaine (10 µM) and/or gp120 IIIB (200 ng/ml) for 15 min and stained with an anti-NF-κB p65 antibody, followed by treatment with an Alexa Flour 488-conjugated secondary antibody. Slides were mounted in Slow Fade antifade reagent (with DAPI, blue nuclear stain) and images were captured by fluorescence microscope. Scale bars indicate 20 µm. (B) Western Blot analysis of nuclear extracts from cocaine and gp120-treated cells for varying times (5 to 60 min) using an antibody specific for p65 subunit of NF-κB. Representative immunoblot and the densitometric analysis of p NF-κB/histone from three separate experiments is presented. All the data in these figures are presented as mean±SD of three individual experiments. *p<0.05 versus control group. (C) Inhibition of NF-κB using the specific inhibitor TPCK (10 µM) resulted in abrogation of cocaine and gp120 toxicity. **p<0.01 versus control group. # p<0.05 versus cocaine + gp120 group.

Activation of the MAPK cascade has been shown to result in translocation of the downstream transcription factor kappa-B (NF-κB) [31]. Nuclear extracts isolated from rat primary astrocytes treated with cocaine and gp120 were examined for the translocation of the p65 subunit of NF-κB using immunocytochemical analysis. As shown in Figure 7A, there was an increased translocation of NF-κB p65 in the nucleus of cocaine and gp120-treated cells versus the untreated control cells. Further validation of these findings was done by Western blot analysis. As shown in the Figure 7B, treatment of astrocytes with both the agents induced time-dependent nuclear translocation of NF-κB p65, an effect that was evident as early as 5 min post-treatment and that was sustained for at least 60 min. To further confirm the role of NF-κB in the toxicity induced by cocaine and gp120, astrocytes cultures were pre-treated with l-1-tosyllamide-2-phenylethyl chloromethyl ketone (TPCK), an inhibitor of NF-κB, followed by treatment of cells with both the agents. Toxicity induced by cocaine and gp120 was significantly abrogated in the presence of the NF-κB p65 inhibitor TPCK (Figure 7C), thus confirming the involvement of NF-κB activation in mediating cocaine and gp120 neurotoxicity.

## Discussion

While astrocyte proliferation is one of the hallmark features of HAD, increasing evidence implies that astrocytic apoptosis could also participate in many neurodegenerative disorders including HAD [14], [32], [33], [34], [35], [36],and furthermore, that astrocytic dysfunction could contribute to neuronal loss and overall pathology [12]. This thinking is plausible since astrocytes provide neurotrophic support, not only by protecting neurons against toxicity induced by excitatory amino acids but also by maintaining normal homeostasis of the extracellular fluid. Hence, keeping in the mind the concept that astrocytic cell death could be induced by HIV env protein, gp120, the present study was undertaken to understand the mechanisms by which cocaine could amplify the toxic response of gp120.

Our findings suggested that cocaine in cooperation with gp120 exacerbated toxicity in rat astrocytic cultures via the apoptotic pathway involving intracellular ROS production, mitochondrial membrane potential loss, and activation of the MAPK signaling pathways and NF-κB. Our results demonstrated that co-treatment of rat primary astrocytes with cocaine and gp120 caused a significant decrease in cell viability compared with cells treated with either agent alone. Apoptosis or programmed cell death is a consequence of concerted activation of proteolytic cascade involving a family of proteases such as caspase-3. To dissect the apoptotic pathway involved in cocaine and gp120-mediated toxicity, activation of caspase-3 was monitored in treated cells using both immunostaining and Western blot analyses. Indeed there was increased activation of caspase-3 in cells treated with both the agents compared with cells treated with either agent alone.

Mitochondrial dysfunction and oxidative stress occur early during the apoptotic pathway and is a common feature of most neurodegenerative disorders. Moreover, there is strong evidence that both mitochondrial dysfunction and oxidative stress are causal factors in the pathophysiology of these disorders [37], [38], [39]. It has been shown that ROS generated in astrocytes can have implications in eliciting oxidative stress to the neighboring neurons [40]. In the present study, co-treatment of rat primary astrocytes with both cocaine and gp120 resulted in enhanced ROS production compared with the astrocytes treated with either agent alone.

Work by various groups has suggested the mitochondria as a link between the initial apoptotic signal and the end point biochemical reactions leading to cell death [41], [42]. Mitochondria play a critical role in the regulation of ROS production and cell death. Mitochondrial dysfunction is thus a prominent feature of apoptosis, and release of pro-apoptotic protein from the mitochondrial intermembrane space has been considered to be a critical event that occurs during apoptosis [43]. In response to death stimuli, mitochondrial membranes become permeabilized, ultimately resulting in activation of initiator and effector caspases [44]. In the present study, co-treatment of primary astrocytes with both cocaine and gp120 resulted in a significant decrease in mitochondrial membrane potential compared with cells treated with either agent alone.

Intracellular signaling mechanisms responsible for gp120 effects in glial cells have not been clearly identified. It has been previously been shown that gp120 can activate the MAPK pathway [45], [46]. MAPK signaling pathways including JNK, p38 and ERK can, in turn, modulate diverse cellular events, such as cell death and survival [47], [48], [49]. Activation of JNK, p38 and ERK pathway in cell death has been well documented [48], [49]. In present study, treatment of astrocytes with cocaine and/or gp120 led to a significant increase in JNK, p38 and ERK/MAPK pathway, thereby underpinning the role of this pathway in cocaine and gp120-mediated apoptosis.

To further validate the role of JNK, p38 and ERK/MAPK signaling pathway in cocaine/gp120-induced astrocytic apoptosis, cells were pretreated with inhibitors specific for JNK (SP600125), p38 (SB203580), and MEK1/2 (U0126) prior to exposure with gp120 and/or cocaine followed by assessment of cell viability. Inhibition of JNK, p38 and ERK/MAPK pathway by their respective inhibitors ameliorated cell toxicity induced by cocaine and gp120.

Next step was to examine the role of downstream mediator of the MAPK pathway, the transcripotion factor NF-κB, which translocates into the nucleus upon activation to initiate transcription of various target genes [50]. Our findings clearly demonstrated enhanced nuclear translocation of NF-κB in astrocytes treated with both cocaine and gp120, and this effect was abrogated in the cells pretreated with the NF-KB inhibitor.

In conclusion, the present study indicates that both cocaine or gp120 elicit similar signaling transduction pathway in astrocytes, involving oxidative stress, mitochondrial membrane potential loss, activation of JNK, p38 and ERK/MAPK pathways and resulting downstream activation of nuclear transcription factor NF-κB. Simultaneous treatment of astrocytes with both the agents, however, leads to an even further activation of the signaling pathways culminating eventually into enhanced astrocytic apoptosis. Such a process could have deleterious ramifications for neuronal survival, especially in areas close to the apoptosing astrocytes. Such a mechanism could have implications for not only HAD, but also other neurodegenerative disorders that have co-morbid association with drugs of abuse. In the central dogma, thus while astrocyte proliferation or astrocytosis is thought of as a major correlate of HAD, it is important to consider contributions of astrocytic death in neuronal survival. Finally, defining the molecular pathways activated in response to drugs of abuse and viral toxins could help pave the way for designing therapeutic strategies aimed at treatment of HAD in drug-abusing population.
